# The population genomic analyses of chloroplast genomes shed new insights on the complicated ploidy and evolutionary history in *Fragaria*

**DOI:** 10.3389/fpls.2022.1065218

**Published:** 2023-02-15

**Authors:** Yanhong Song, Chaochao Li, Lifeng Liu, Panpan Hu, Gang Li, Xia Zhao, Houcheng Zhou

**Affiliations:** ^1^ Zhengzhou Fruit Research Institute, Chinese Academy of Agricultural Sciences, Zhengzhou, China; ^2^ Key Laboratory of Horticultural Plant Biology (Ministry of Education), Huazhong Agricultural University, Wuhan, China

**Keywords:** *Fragaria*, chloroplast genome assembly, population structure, diversity, evolutionary

## Abstract

The genus *Fragaria* consists of a rich diversity of ploidy levels with diploid (2x), tetraploid (4x), pentaploid (5x), hexaploidy (6x), octoploid (8x) and decaploid (10x) species. Only a few studies have explored the origin of diploid and octoploid strawberry, and little is known about the roles of tetraploidy and hexaploidy during the evolution of octoploid strawberry. The chloroplast genome is usually a stable circular genome and is widely used in investigating the evolution and matrilineal identification. Here, we assembled the chloroplast genomes of *F*. x *ananassa* cv. ‘Benihoppe’ (8x) using Illumina and HiFi data seperately. The genome alignment results showed that more InDels were located in the chloroplast genomes based on the PacBio HiFi data than Illumina data. We obtain highly accurate chloroplast genomes assembled through GetOrganelle using Illumina reads. We assembled 200 chloroplast genomes including 198 *Fragaria* (21 species) and 2 *Potentilla* samples. Analyses of sequence variation, phylogenetic and PCA analyses showed that *Fragaria* was divided into five groups. *F*. *iinumae*, *F*. *nilgerrensis* and all octoploid accessions formed Group A, C and E separately. Species native to western China were clustered into Group B. Group D consisted of *F*. *virdis*, *F*. *orientalis*, *F*. *moschata*, and *F*. *vesca*. STRUCTURE and haplotype network confirmed that the diploid *F*. *vesca* subsp. bracteata was the last maternal donator of octoploid strawberry. The dN/dS ratio estimated for the protein-coding genes revealed that genes involved in ATP synthase and photosystem function were under positive selection. These findings demonstrate the phylogeny of totally 21 *Fragaria* species and the origin of octoploid species. *F*. *vesca* was the last female donator of octoploid, which confirms the hypothesis that the hexaploid species *F*. *moschata* may be an evolutionary intermediate between the diploids and wild octoploid species.

## 1 Introduction

Chloroplasts are organelles with seni-autonomous genetic systems, playing a vital role in energy converters for higher plants (Neuhaus and Emes, 2000). Compared with the nuclear genome, the plant chloroplast genomes have relative conservation in structure composition and gene type (Wu et al., 2011; Bock and Knoop, 2012; Dong et al., 2012; Dong et al., 2013; Liang et al., 2020). The chloroplast genomes are ideal for studying plant phylogenetic analysis and species identification due to their simple structure, lack of recombination, and uniparental inheritance characteristics (Clegg et al., 1994; Ahmed et al., 2013; Mower and Vickrey, 2018). The first chloroplast genome (*Nicotiana tabacum*) was sequenced in 1986 (Shinozaki et al., 1986). As the sequencing technology develops, there has been a sharp increase in the number of chloroplast genomes from cereals, fruits, vegetables, and other flowering plants (Maier et al., 1995; Schmitz-Linneweber et al., 2001; Wu et al., 2010; Ruhfel et al., 2014; Yan et al., 2022). Comparative chloroplast genomes of *Gossypium*, *Atractylodes*, *Musa*, *Medicago*, *Citrus*, and other species were conducted to reveal genetic variation, phylogenetic relationship, and plastome evolution (Xu et al., 2013; Carbonell-Caballero et al., 2015; Zhang et al., 2020; Wang et al., 2021; Brock et al., 2022; Jiao et al., 2022; Li et al., 2022; Song et al., 2022; Yisilam et al., 2022). Combined with the nuclear genomes, chloroplast genomes were used to explore the haplotype development and phylogenetics relationship of *Japanese apricot* from different geographical locations (Huang et al., 2022).

Genome assembly toolkits and sequencing reads are two keys to accurate genomes. Next-generation sequencing (NGS) methods became an effective approach to producing chloroplast genome sequences after Sanger sequencing. However, short-read produce large amounts of DNA fragments ranging from 50-400 bp, which makes it challenging to assemble accurate genomes, especially for repeat-rich samples. The long-reads of third-generation sequencing (TGS), such as Oxford Nanopore Technologies (ONT) ultra-long reads and Pacific Biosciences (PacBio) highly accurate long read (HiFi), delivers even up to 200 kb long reads (Istace et al., 2017). Circular consensus sequencing (CCS) reads are long reads with a low error rate, meaning they allow the assembly of repeated regions. CCS reads were used to accurately assemble and detect SNPs of chloroplast genomes (Li et al., 2014). Although there is no systematic comparison between the HiFi CCS and short-read for assembling chloroplast genomes, the quality expectation for such small but important genomes is as high as complete and accurate in the community. Specifically, whether and how HiFi reads could be used to generate high-quality chloroplast genomes is untested. GetOrganelle is an efficient and accurate toolkit for *de novo* assembly of organelle genomes (Freudenthal et al., 2020; Jin et al., 2020; Odago et al., 2021; Ruang-Areerate et al., 2021; Singh et al., 2021; Drown et al., 2022; Liu et al., 2022; Zhao et al., 2022). It can assemble better plastomes using low coverage WGS data compared with NOVOplasty. Thus, we chose GetOrganelle to obtain accurate chloroplast genomes as a reference to correct genome sequences based on CCS reads.

The genus *Fragaria* includes ~25 identified species and comprises natural ploidy levels consisting of diploids (2n =14), tetraploids (4n =28), pentaploids (5n =35), hexaploids (6n =42), octoploids (8n =56) and decaploids (10n =70) (Staudt, 1962; Hummer et al., 2009; Staudt, 2009). Previous genome studies have focused on diploids and octoploids (Shulaev et al., 2011; Hardigan et al., 2019; Edger et al., 2019; Feng et al.,2021; Qiao et al., 2021), the origin and evolution of tetraploid and hexaploid strawberry remain unknown. China has been a distribution center of *Fragaria* resources for fourteen of twenty-five species spread in northeastern, northwestern, and southwestern China (Deng and Lei, 2005; Lei et al., 2017). The tetraploid strawberry includes five species: *F*. *orientalis*, *F*. *moupinensis*, *F*. *corymbosa*, *F*. *gracilis*, and *F*. *tibetica*. Except for *F*. *orientalis*, other species are native to China. *Fragaria moschata* (musk strawberry or hautbois strawberry) is native to Central Europe but has been replaced by *F*.x *ananassa* at the end of the 19th century (Darrow, 1966). The tetraploid and hexaploid strawberry are dioecious (individuals are either females or males) and unique characteristics in fruit aroma and resistance. *F*. *moupinensis* has strong adaptability and disease resistance (Guo et al., 2018). *Fragaria moschata* resists diseases like bacterial angular leaf spot disease (Maas et al., 1995) and powdery mildew (Kantor, 1984). They are cultivated commercially for their intense aroma and flavor (Kantor, 1984).

The cultivated strawberry (*Fragaria* x *ananassa*) is a young species formed less than 300 years ago through a spontaneous hybridization between the allo-octoploid species *Fragaria virginiana* and *Fragaria chiloensis* (Darrow, 1966; Given et al., 1988; Staudt, 2009). It spread globally from France for its fruit flavor and juicy flesh. It is an allo-octoploid species (2n =8× = 56), composed of 56 chromosomes organized in four diploid progenitor species. Previous phylogenetic studies reported that octoploid genomes consisted of four or five diploid progenitors (Fedorova, 1946; Tennessen et al., 2014; Kamneva et al., 2017; Yang and Davis, 2017; Edger et al., 2019; Liston et al., 2020; Feng et al., 2021). *F*. *vesca* and *F*. *iinumae* as two of the diploid progenitor species have been identified, the other species are still controversial. Based on a near-complete chromosome-scale assembly for octoploid strawberry ‘Camorosa’, phylogenetic analyses provided genome-wide support for the two unknown progenitors: *F*. *virids* and *F*. *nipponica* (Edger et al., 2019). According to the geographical distribution, tetraploid and hexaploid species may be involved in the evolution of octoploid strawberry (Edger et al., 2019). However, Liston et al., found no support for *F*. *virids*, *F*. *nipponica* and *F*. *moschata* as ancestors (Liston et al., 2020). Research also drawn a conclusion that *F*. *viridis* was not the diploid progenitor (Feng et al.,2021) using sppIDer (Langdon et al., 2018). In summary, the cytoplasm donor of wild strawberry remains unknown.

For the present study, we assembled the chloroplast genomes of *F*. x *ananassa* cv. ‘Benihoppe’ based on Illumina and CCS reads. Next, we sequenced 33 samples, including tetraploid species (*F*. *orientalis*, *F*. *moupinensis*, *F*. *corymbose*, *F*. *gracilis*, *F*. *tibetica*) and hexaploidy species (*F*. *moschata*) with Illumina HiSeq X Ten platform. We collected a total of 200 illumina data including NCBI database source, consisting of twenty-one *Fragaria* species and *Potentilla*. With the GetOrganelle toolkit, we obtained 165 complete circular chloroplast genomes successfully. Our main objects were to (1) compare the chloroplast genomes assembled with long- and short-read data; (2) conduct population genomic analyses of chloroplast genomes of *Fragaria* genus; (3) shed new insights on the population constructure and evolutionary history of strawberry. These results provided new insights into *Fragaria* species cluster and the origin of octoploid strawberry.

## 2 Materials and methods

### 2.1 Plant material and DNA sequencing

We conducted a comparison of chloroplast genomes of *F*. x *ananassa* cv. ‘Benihoppe’ assembled with long- and short-read data. In order to perform the test, we extracted DNA of the young leaves of ‘Benihoppe’ for the construction of CCS libraries and Illumina short-read libraries and sequenced them on the PacBio Sequel and Illumina HiSeq X Ten platform reapectively. A total of 10 Gb of HiFi reads and 20 Gb of Illumina reads were generated for the assembly of chloroplast genomes.

A total of 200 Illumina sequences were examined in this study. Of these, Illumina paired-end sequences of 167 accessions were downloaded from the NCBI Sequence Read Archive database (https://www.ncbi.nlm.nih.gov/sra) (**Table S2**). The rest of 33 *Fragaria* accessions, including *F*. *orientalis*, *F*. *moupinensis*, *F*. *corymbose*, *F*. *gracilis*, *F*. *tibetica* and *F*. *moschata*, were newly sequenced. The Illumina sequence are available in the NCBI SRA (BioProject ID: PRJNA913463). The fresh and young leaves of *Fragaria* accessions were collected from Zhengzhou Fruit Research Institute, CAAS. Extraction of the whole genomic DNA from fresh leaves of these species was performed with a modified Cetyltrimethylammonium bromide (CTAB) method. A 150 bp of paired-end libraries were constructed and PE150 sequencing was performed on the Illumina HiSeq X Ten platform.

### 2.2 Chloroplast genome assembly and annotation

We used Fastp software (v0.20.1) to filter the low-quality reads of 200 next-generation sequencing data, and then assembled by GetOrganelle (v 1.7.6) pipeline (Jin et al., 2020) with the optimized parameters “-fast -k 65,105,127 -w 0.68 -t 10 -f embplant_pt”. We obtained Illumina short-reads and PacBio HiFi sequences for *Fragaria* x *ananassa* cv. ‘Benihoppe’ to compare and correct the chloroplast genomes. About 10 Gb CCS clean reads were used to assemble the ‘Benihoppe’ genome contigs with the default parameters with Canu (v2.2, Koren et al., 2017) and hifiasm (v 0.15.5-r 350, Cheng et al., 2021). The chloroplast genome contigs based on HiFi reads were selected from the Blast using the ‘Benihoppe’ chloroplast genome based on short-reads as a reference. Three different hifiasm_contigs and canu_contigs could cover the chloroplast genome separately. Comparison of pair-wise alignment of ‘Benihoppe’ chloroplast genomes was performed by the mVISTA (http://genome.lbl.gov/vista/mvista/submit.shtml) with the Shuffle-LAGAN mode. Sequences of ‘Benihoppe’ chloroplast genome based on short-reads were used as a reference.

We obtained 165 complete chloroplast genome sequences. All circular chloroplast genomes were annotated with the PGA (Qu et al., 2019) and GeSeq (Tillich et al., 2017) (https://chlorobox.mpimp-golm.mpg.de/geseq.html), by using five GenBank-formatted file of *F.* x *ananassa* (MZ851773, KY358226), *F*. *vesca* (JF345175), *F. orientalis* (NC_035501) and *F*. *moschata* (MW537852) as the database. By comparing the annotation results and removing the incorrect annotations, we obtained 85 protein-coding genes (PCGs), 37 tRNAs and 8 rRNAs.

### 2.3 Mapping, variant calling and annotation

Clean reads of 200 next-generation sequencing data were then separately mapped to the ‘Benihoppe’ chloroplast genome using Burrows-Wheeler Aligner (BWA) software (v0.7.17-r1188) (Li and Durbin, 2009). Alignment files were converted SAM (Sequence Alignment Map) files into sorted BAM (binary version of SAM) files with SAMtools (v1.11). Then, the removal of duplicates was performed using Picard Tools (v2.27.4). Finally, the Variant Call Format (VCF) was obtained with Deepvariant (rc1.0.0). The GVCF files from 200 accessions were consolidated into a single VCF file using GLnexus (v1.2.7). The VCF file was used to annotate mutation sites using the software snpEff (v5.1) (Cingolani et al., 2012).

### 2.4 Phylogeny and population structure analyses

We constructed phylogenetic trees using a maximum likelihood-based method. Vcftools (v0.1.16) was used to extract sequence variation data from VCF files. The phylogenetic tree was conducted by IQ-TREE (v2.1.2) (Nguyen et al., 2015) with the ‘GTR + I+G’ model and 1000 bootstrap replicates based on sequence variation. Based on the evolution relationship and ploidy level within 198 samples, we defined six subgroups in diploid species and three subgroups in tetraploid species for further analysis (*F*. *iinumae* as 2x-1, *F*. *nubicola* and *F*. *nipponica* as 2x-2, *daltoniana* and *F*. *chinensis* as 2x-3, *F*. *nilgerrensis* as 2x-4, *F*. *virdis* as 2x-5, *F*. *vesca*, *F. mandschurica* and *F.bucharica* as 2x-6; *F*. *moupinensis* and *F*. *tibetica* as 4x-1, *F*. *corymbose* and *F*. *gracilis* as 4x-2, *F*. *orientalis* as 4x-3*).*

**Figure 1:**
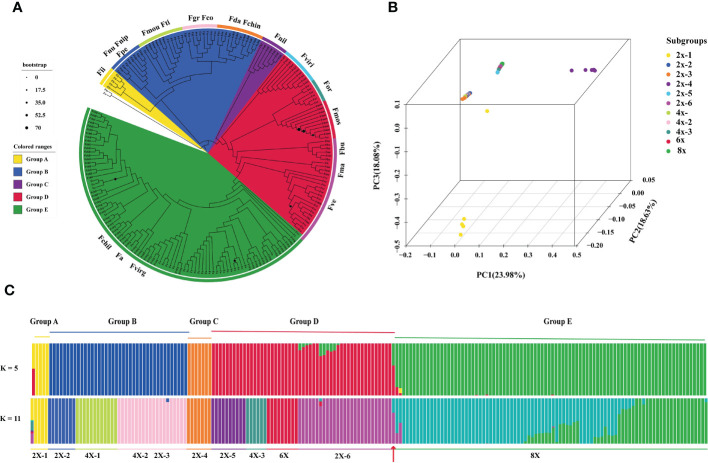
Analysis of Fragaria population genetic structure. **(A)** Phylogenetic tree based on sequence divergence of chloroplast genomes, with the colors of each branch region indicating the different groups (group A, B, C, D, E and outgroup). The colored circle outside the specie names represented the eleven subgroups: 2x-1, Fii, *F iinumae*; 2x-2, Fnu, *F nubicola*, Fnip, *F nipponica*, Fpe, *F pentaphylla*; 2x-3, Fda, *F daltoniana*, Fchin, *F chinensis*;2x-4, Fnil, *F nilgerrensis*; 2x-5, Fviri, *F viridis*; 2x-6, Fbu, *F bringhurstii*; Fma, *F mandschurica*; Fve, *F vesca*; 4x-1, Fmou, *F moupinensis*; Fti, *F tibetica*; 4x-2, Fgr, *F gracilis*; Fco, *F corymbosa*; 4x-3, *F orientalis*; 6x, Fmos, *F moschata*;8x, Fvirg, *F virginiana*; Fchil, *F chiloensis*; Fa, *F*.x *ananassa*. The blue circle sizes are shown as the percentage of bootstrap support less than 70%; **(B)** Principal component analysis (PCA) plot of the 198 *Fragaria* accessions, PC1, PC2 and PC3 explained 23.98%, 18.64% and 18.08% proportion of variance; **(C)** Population stratification analyses of Fragaria species. ADMIXTURE plots for representative *Fragaria* accessions and the outgroup for K= 5 and K= 11. The order of *Fragaria* species was in line with the phylogenetic tree. The red arrow indicates the diploid *F vesca* subsp. bracteate.

To analyze the population structure of chloroplast genomes in *Fragaria*, we conducted the principal component analysis (PCA) for 198 *Fragaria* accessions with filter SNPs using Plink (v1.9) pipeline (Purcell et al., 2007). Then, we conducted the ADMIXTURE (v1.3.0) (Alexander et al., 2009) to estimate the genetic ancestry of 198 *Fragaria* samples. The K=2 to 12 hypothetical ancestral populations were formed, and k=5 and k=11 is shown in **Figure 1**.

### 2.5 Genetic differentiation and population gene selection and haplotype

For the assessment of genetic differentiation and sequence divergence of the *Fragaria* population, we performed a sliding windows analysis to compute the F-statistics (*F_st_*) and nucleotide diversity (π) based on the sequence variation as recommended by genomics_general (https://github.com/simonhmartin/genomics_general). The nucleotide diversity (π) should only be computed within species. We clustered the subgroups by their extremely close relationship using a phylogenetic tree. We calculated π over multiple species in a subgroup for its minor sequence divergence.

To study the molecular evolution of twenty-two *Fragaria* species, the patterns of synonymous (dS), nonsynonymous (dN) nucleotide substitutions and the ratio of nonsynonymous to synonymous rates (dN/dS) were calculated in PAML (v4.10.5) using the CODEML option with codon frequencies estimated using the F3 × 4 model, after removing the duplicated gene and the stop codon of the gene. We conducted a haplotype network for all *Fragaria* species using POPART (v1.7.1) (Leigh & Bryant, 2015) to calculate the gene flow diversity of haplotypes.

## 3 Results

### 3.1 *De novo* chloroplast genome assembly based on short- and long-read data

GetOrganelle was used to accurately assemble the chloroplast genomes of *Fragaria* x *ananassa* cv. ‘Benihoppe’. With the rapid development of high-throughput sequencing technologies, it is feasible to assemble complete chloroplast genomes using the low-coverage whole-genome sequencing data. Following the decreased HiFi sequencing costs in recent years, we want to know if HiFi reads could be used to generate high-precision chloroplast genomes. First, the Illumina data of ‘Benihoppe’ ranging from 1G to 10G was used to test the lowest necessary sequencing coverage to assemble complete chloroplast genomes with GetOrganelle. The results showed that 2G reads were enough to get the circular chloroplast genomes, and a further increase in the sequencing data did not improve the genomes further (Wang et al., 2018; Jin et al., 2020).

**Figure 2:**
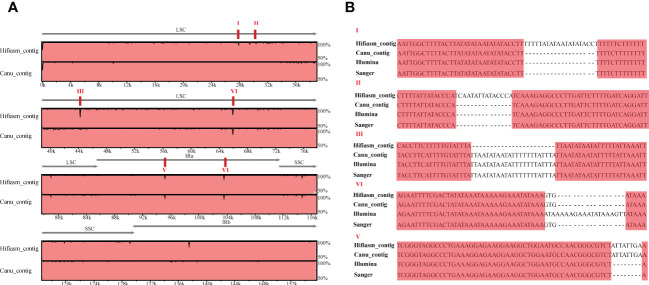
Visualized alignment and identity percent among the *Fragaria* chloroplast genomes based on three assembly methods relying on short- and long-read sequencing. **(A)**The figure was generated using mVISTA. The visible “peaks and valleys” graph shows the pairwise sequence alignement identity with the Benihoppe chloroplast genome assembled using Illumina data. The top and bottom percentages are displayed to the right of every row. The red boxes indicate six positions of identified InDels; **(B)** The verification of PCR amplification and Sanger sequence of the obvious InDels in A.

Furthermore, we conducted chloroplast genome assembly using HiFi data of the same cultivated strawberry ‘Benihoppe’. About 10 Gb of CCS clean reads were used to assemble the primary contigs with default parameters of Hifiasm (v 0.15.5-r 350) and Canu (v 2.2). Taking the chloroplast genome assembled by short-read as a reference, three contigs can overlap the whole genome (**Figure S1**). Visualized alignment of the three versions of chloroplast genomes (v1_Illumina, v2_Hifiasm_contig, and v3_Canu_contig) sequences using mVISTA (Frazer et al., 2004). Each horizontal row represents the pairwise sequence alignment identity percent. Compared with two versions of long-read genomes, the genome based on Canu-contigs has more SNP and minor InDels (especially the 34-46 kb region). Seven and four obvious InDels were found in Hiasm-contigs and Canu-contigs. Verification through PCR amplification and Sanger sequencing (primers are shown in **Table S1**) was used to verify the differences between the three versions. Chloroplast genomes with short-read data have much higher accuracy in InDels except for those located around 66kb (aligned sequences were shown in **Figures 2**, **S2–7**). We manually corrected the sequences of chloroplast genomes assembled using Illumina reads by GetOgranelle, and taked the genome as a reference for further analysis.

By comparing the annotation resulted by PGA and Geseq, and removing the incorrect annotations, eighty-five protein-coding genes (PCGs), 37 transfer RNA (tRNA) genes, and 8 ribosomal RNA (rRNA) genes were predicted in the *F.* x *ananassa* cv. ‘Benihoppe’.

### 3.2 Phylogenetic analyses and structure of strawberry species

**Figure 3:**
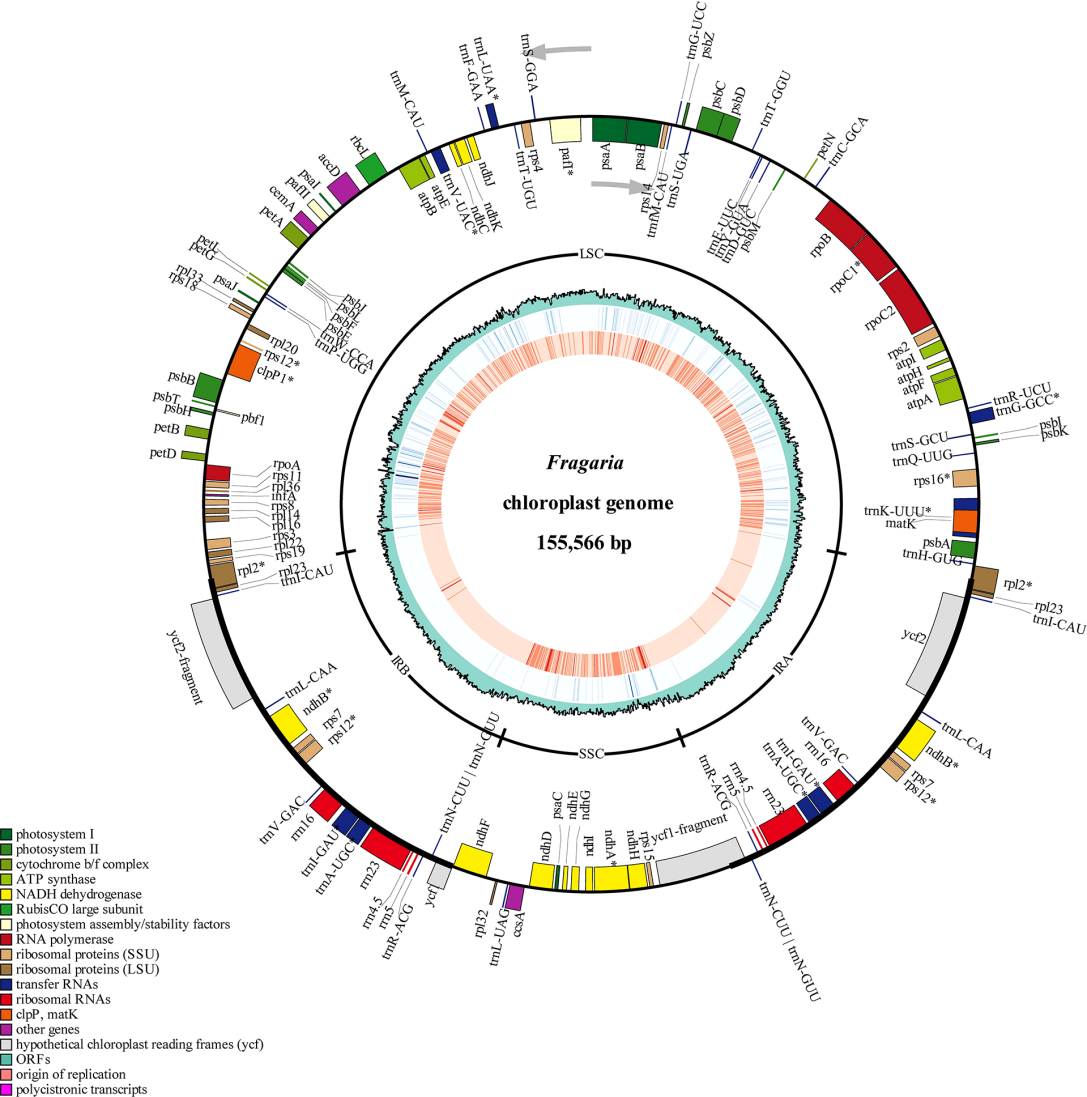
Chloroplast genome map of 198 *Fragaria* accessions. The gene position, quadripartite structure, GC content, density of InDels and SNP distribution were shown from the outer to inner rings. The outermost rectangles represented annotate genes belonging to different functional groups. Gene blocks on the outside and inside the circle indicated the clockwise and anticlockwise transcribed genes, respectively.

In order to apply the assembly methods to the genetic study of strawberry population, we assembled 165 complete circular chloroplast genomes from the 200 samples using GetOrganelle (**Figure 3**; **Table 1**). The average length of the plastid genomes is 155,644 bp. The genome sizes among complete genomes ranged from 155,493 bp for *F*. *viridis* to 155,809 bp for *F*. *hayatae*. The GC contents were 37.18% (*F. iinumae*) - 37.29% (*F*. *viridis*).

**Table 1:** Chloroplast genome features of 22 *Fragaria* species (21 wild species and 1 cultivated strawberry).

Ploidy	Species	Category	Number of samples	Number of complete genomes	Average complete genome size (bp)	Average GC content (%)
2X	*F. iinumae*	2X- 1	5	2	155621.00	37.18
	*F. nubicola*	2X-2	4	3	155681.67	37.24
	*F. nipponica*	2X-2	4	1	155689.00	37.24
	*F. pentaphylla*	2X-2	2	2	155671.00	37.25
	*F. daltoniana*	2X-3	3	2	155638.00	37.25
	*F. chinensis*	2X-3	7	4	155645.25	37.25
	*F. nilgerrens*	2X-4	8	5	155760.20	37.26
	*F. viridis*	2X-5	10	10	155492.70	37.29
	*F. bucharica*	2X-6	4	2	155588.50	37.25
	*F. mandschurica*	2X-6	6	4	155572.00	37.25
	*F. vesca*	2X-6	14	8	155659.25	37.23
	*F*. x *bifera*	2X-6	2	2	155701.00	37.22
4X	*F. moupinensis*	4X-1	6	5	155667.80	37.25
	*F. tibetica*	4X-1	6	6	155664.83	37.24
	*F. gracilis*	4X-2	5	5	155673.00	37.25
	*F. corymbosa*	4X-2	6	5	155658.20	37.24
	*F. orientalis*	4X-3	7	7	155600.00	37.24
6X	*F. moschata*	6X	9	8	155625.00	37.24
8X	*F. virginiana*	8X	38	37	155581.78	37.24
	*F.* x *ananassa*	8X	30	28	155585.86	37.24
	*F. chiloensis*	8X	22	19	155584.76	37.23

A comparison of the chloroplast genomes within *Fragaria* species showed that the sequence is highly conserved. Taking the chloroplast genome of ‘Benihoppe’ assembled with short-read data as reference. Among the 200 accessions, 4,551 single nucleotide polymorphisms (SNPs) and 621 small insertions and deletions (InDels) were identified. To further explore the roles of tetraploid and hexaploid species in polyploid formation, the phylogenetic trees were inferred using sequence variation of 198 accessions, with the genus *Potentilla* as the outgroups (**Figure 1A**). All Fragaria species could be divided into five groups. *F*. *iinumae*, the oldest extant species, and *F*. *nilgerrensis* formed a single Group A and Group C, separately. Group B included *F*. *nubicola*, *F*. *nipponica*, *F*. *moupinensis* (4x), *F*. *tibetica* (4x), *F*. *gracilis* (4x), *F*. *corymbosa* (4x), *F*. *daltoniana* (2x), and *F*. *chinensis* (2x). Group B contained four tetraploid species, in agreement with previous phylogenetic analyses using chloroplast sequences (Potter et al., 2009; Njuguna et al., 2013). In previous studies, *F*. *pentaphylla* (2x) and *F*. *nubicola* (2x) were supposed to be the progenitors of *F*. *moupinensis* (4x) and *F*. *tibetica* (4x) by target capturing sequence (Kamneva et al., 2017). *F*. *corymbosa* (4x) might be originated from *F*. *chinensis* (2x) for geographical distribution and similarity in morphological traits (Staudt, 2009). From our phylogenetic tree, tetraploid *F*. *corymbosa* (4x), *F*. *gracilis* (4x), and diploid *F*. *chinensis* (2x) are in the same clade, and *F*. *chinensis* (2x) might not be the female donator. The diploid species *F*. *virdis* (2x) was sister to the tetraploid species *F*. *orientalis* (4x-3) and hexaploid species *F*. *moschata* in group D. Meanwhile, *F*. *vesca* subsp. bracteata was the latest diploid donator to *F* x *ananassa*.

**Figure 4:**
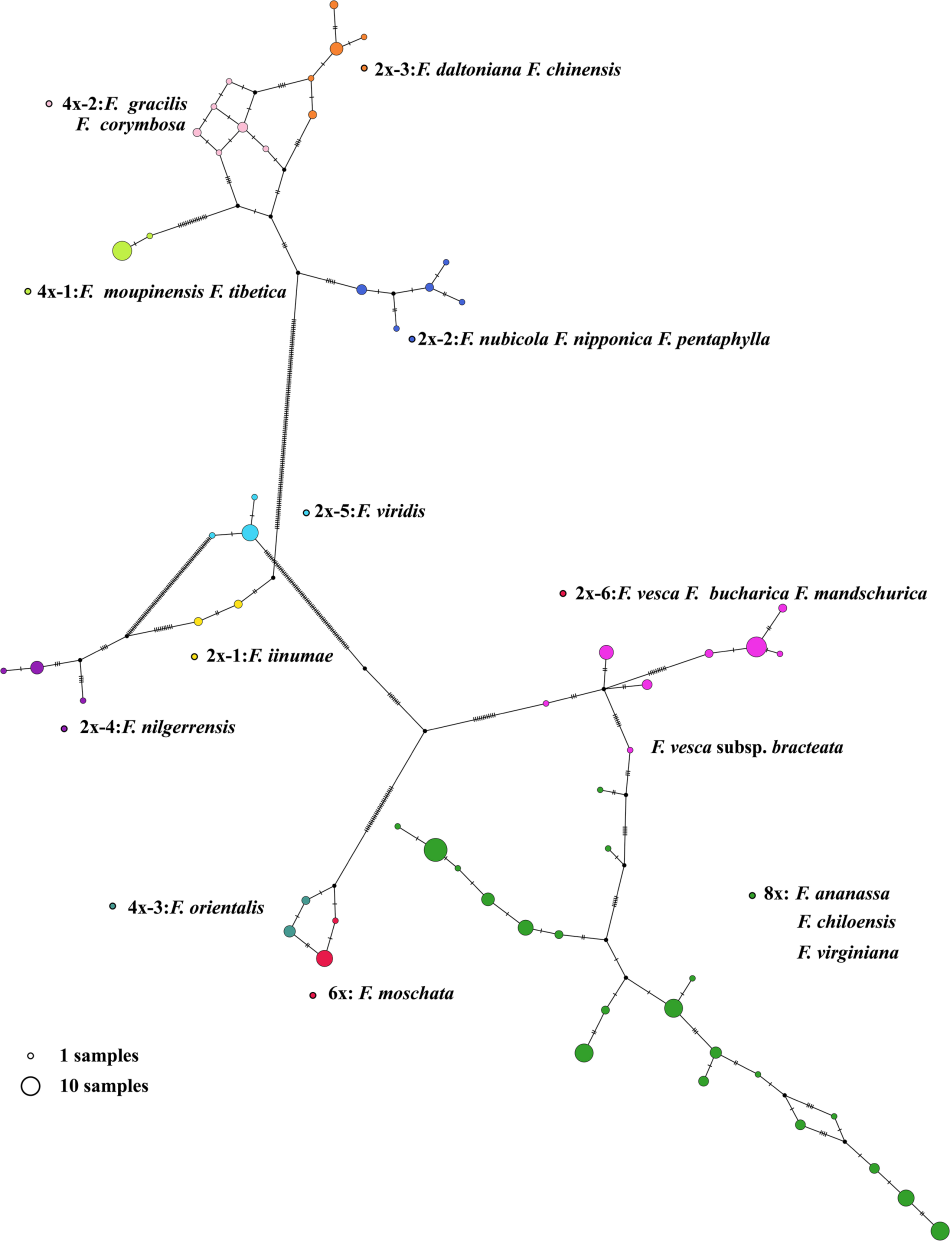
The chloroplast haplotypes network of Fragaria species. The size of the circle represents the number of haplotypes. Dots represent putative haplotypes. Mutations are represented by perpendicular dashes. The red arrow indicates the diploid *F*. *vesca* subsp. bracteate.

We conducted principal components analysis (PCA) to visualize the relationships between the *Fragaria* samples (**Figure 1B**). The *Fragaria* species formed four groups: group A (2x-1), group B and group C (2x-4), forming three distinct groups in accord with the clustering results of phylogenetic trees. Group D and E clustered together. Further, we applied the ADMIXTRUE analysis to all the samples based on sequence divergence. With K=5, *Fragaria* species groups were in line with the taxa of the phylogenetic tree. Haplotype analyses showed specific proof that k=5 could be distinguished from 198 accessions of 21 species (**Figure 4**). *F*. x *ananassa* had a mixture of *F*. *vesca* subsp. bracteate, suggesting recent introgression between these two species (**Figure 1C**). When K=11, it was notable that *F.* x *ananassa* originated from natural hybridization between *F*. *chiloensis* and *F*. *virginiana*.

### 3.3 Genetic diversity within *Fragaria* species

**Figure 5:**
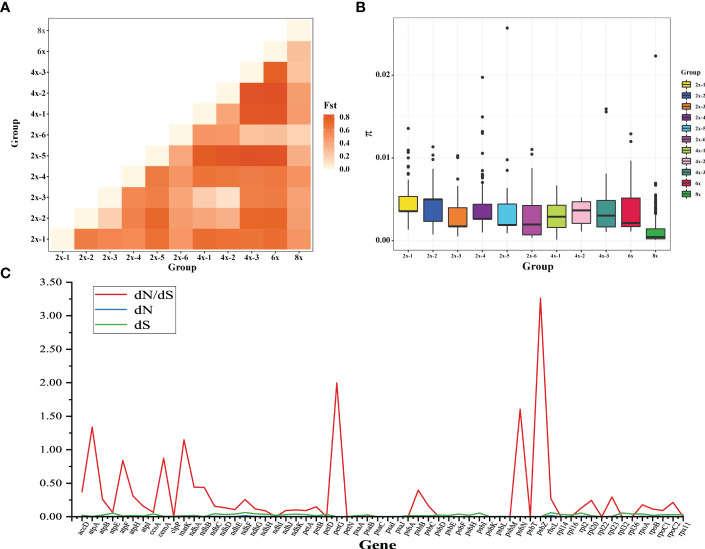
Analysis of Fragaria population genetic structure. **(A)** the pairwise Fst values between species of different ploidy levels; **(B)** Nucleotide diversity of chloroplast genome sequences of *Fragaria* species; **(C)** The estimations of the ratio of nonsynonymous to synonymous rates (dN/dS) of plastid protein-coding genes (PCGs).

To further analyze the relationship among species, we defined *F*. *moupinensis* (4x), *F*. *tibetica* (4x), as 4x-1, and *F*. *corymbose* (4x), *F*. *gracilis* (4x) as 4x-2. All *Fragaria* species were divided into 11 subgroups in **Table 1**. We calculated differentiation values (Fst) across all pairwise taxa comparisons. Lower Fst values were found between taxa in the same groups. For example, the overall lowest Fst was observed between 4x-1/2 and 2x-2/3. Among tetraploid species, 4x-3 shows a high Fst value compared to 4x-1 and 4x-2. Results are consistent with PCA analysis (**Figure 1B**). However, overall lowest Fst was obtained between 8x and other species (**Figure 5A**).

The nucleotide diversity (π) was used to assess the level of sequence divergence in the chloroplast genomes of *Fragaria* species. The value of π showed that the lowest nucleotide diversity was found in octoploid accessions, even though 8x has more accessions than others in our study (**Figure 5B**). *F*. *virdis* (2x-5) underwent more mutation to 4x-3, 6x, and 2x-6. We couldn’t infer their ancestors from the absence of samples (extinct or uncollected progenitors), although they share the same clade in the phylogenetic tree. We also calculated the dN/dS ratio of protein-coding genes in the *Fragaria* chloroplast genomes (**Figure 5C**). The average dN/dS ratio of the 72 common protein-coding genes studied in the genomes was 0.27. The protein accD, matK, petG, psbN, and psbZ were under positive selection due to dn/ds ratio above 1. These genes involve in ATP synthase and photosystem function.

## 4 Discussion

### 4.1 Assembly of chloroplast genomes of *Fragaria* species

Strawberry genus, *Fragaria* L. includes ~25 identified species and comprises natural ploidy levels ranging from diploid (2n =14) to decaploid (10n =70), making it a research model for studying ploidy variations. Previously, the chloroplast genomes of 25 accessions representing 21 *Fragaria* species were assembled using genomic DNA and PCR pool sequencing, with 49% - 99% completeness (Njuguna et al., 2013). Twenty-seven (Li et al., 2021) and ten (Sun et al., 2021) *Fragaria* species were sequenced and obtained a chloroplast genome size of 155,479~155,832bp and 155,459~155,705 bp, respectively. In this study, we assemble the chloroplast genomes of *F*. x *ananassa* cv. ‘Benihoppe’ using short- and long-read data. Previous studies have assembled chloroplast genomes using PacBio and ONT data (Ferrarini et al., 2013; Wu et al., 2014; Redwan et al., 2015). After error correction, PacBio sequence data has an advantage on generating complete genome assemblies. A circular consensus sequencing (CCS) strategy was applied to assemble accurate genomes (Li et al., 2014).

Nevertheless, there is still a dearth of knowledge about the best approach to obtain accurate genomes. Here, we compared the genomes between short- and long-read data. The alignment results showed that chloroplast genomes based on hifiasm_contigs detected more InDels than canu_contigs (**Figure 2**). We used PCR to amplify target DNA segments, including InDels, to check which assembly was the most accurate. We found that the chloroplast genome of ‘Benihoppe’ using GetOrganelle with Illumina data was the most highly accurate genome assembly. To conclude, we suggest using short read Illumina data for chloroplast genome studies.

In our study, 165 new complete circular chloroplast genomes were obtained from 200 samples within 21 *Fragaria* species were obtained using the GetOrganelle toolkit, with an average of 155,644 bp in length (**Figure 3**). The chloroplast genomes of plants have highly conserved structures, with a quadripartite structure including two copies of an IR region and large and small single-copy (LSC and SSC) regions. Although several studies have revealed variation and evolution of the whole chloroplast genomes, to the best of our current knowledge, the accession number we assembled represents a more comprehensive analysis to date. As the sequencing technology and toolkits develop, it is possible to obtain complete and accurate chloroplast genomes. Based on the *Fragaria* population here, the results bring light to the origin and diversity of the genus. For example, haplotype analysis showed that *F*. *vesca* subsp. bracteata is the direct maternal source of octoploid strawberry (**Figure 4**).

### 4.2 Diversity and phylogenetic studies

Earlier phylogenetic analyses relied on the DNA sequence of partial chloroplast genomes or several genes (Jansen et al., 2007). Even though the use of DNA fragments enhanced the analysis, there are still uncertainties in information in the taxa relationship. Complete chloroplast genome sequences are valuable for phylogeny group classification and evolution of plant species. Based on pollen morphology and distribution, A Eurasian-American *Fragaria* group included six diploid, one tetraploid, one hexaploidy, and all octoploid species (Staudt, 2009). The classification is consistent with previous phylogenetic studies (Njuguna et al., 2013; Li et al., 2021; Sun et al., 2021). *F*. *nilgerrensis* species contains two subspecies *nilgerrensis* and *hayatae*. In our phylogenetic analysis, *F*. *nilgerrensis* forms an independent group C. The PCA results also showed that *F*. *nilgerrensis* separates from the rest *Fragaria* species (**Figure 1B**). However, it is uncertain that *F*. *nilgerrensis* was placed as a sister to *F*. *chinensis* or *F*. *virdis* in the previous chloroplast genomes and nuclear genomes analysis (Potter et al., 2009; Rousseau-Gueutin et al., 2009; Qiao et al., 2021). Chloroplast capture resulting from hybridization may explain the discordance between trees based on chloroplast DNA and nuclear genes (Soltis and Kuzoff, 1995). Chloroplast haplotype analysis showed that *F*. *nilgerrensis* was closely associated with *F*. *iinumae*. *F*. *nilgerrensis* is a widely distributed diploid strawberry native to southwest China and provides valuable genetic variations for breeding. This species is very different from other species and is easy to identify. The decaploid strawberry cultivar ‘Tokun’ origined from the hybridization between *F*. *nilgerrensis* and *F*. x *ananassa* (Noguchi, 2011). The evolutionary relationships of *F*. *nubicola*, *F*. *pentaphylla*, *F*. *moupinensis*, *F*. *tibetica*, *F*. *corymbosa*, *F*. *gracilis*, *F*. *chinensis*, and *F*. *daltoniana* have never been revealed (Rousseau-Gueutin et al., 2009; Li et al., 2021). These species, clustered into group B, are limited in distribution in Western China (Lei et al., 2017). In our results, *F*. *nubicola* and *F*. *pentaphylla* are sisters to the *F*. *moupinensis* and *F*. *tibetica* with 100% bootstrap support (**Figure 3**). Taking into account the overlapping geographical distribution in Southwest China and similar morphological characteristics of *F*. *pentaphylla*, *F*. *moupinensis*, and *F*. *tibetica*, *F*. *pentaphylla* may be a common female parent of tetraploid species *F*. *moupinensis* and *F*. *tibetica* (Rousseau-Gueutin et al., 2009; Kamneva et al., 2017; Li et al., 2021).

In our study, 4x-2 (*F*. *corymbose* and *F*. *gracilis*) and 2x-3 (*F*. *chinensis* and *F*. *daltoniana*) are sister species, and these species are distributed in Northwest China. Moreover, the morphological characteristics of *F*. *corymbose* and *F*. *gracilis* are similar in runners, petioles and calyx. Our phylogenetic analysis also supported that *F*. *corymbosa* and *F*. *gracilis* may share the same ancestor (Rousseau-Gueutin et al., 2009). Staudt et al. (2009) pointed out *F*. *chinensis* may be one of the ancestors of *F*. *corymbose*. Combined with the results of the phylogenetic tree and haplotype network, *F*. *nubicola* and *F*. *pentaphylla* may be the ancestors of *F*. *moupinensis*, *F*. *tibetica*, *F*. *corymbosa*, *F*. *gracilis* for sharing the overlapping geographical distribution. More samples need further research to explore the origin and evolution of these species.

*F*. *virdis* belonged to group D and was a sister to *F*. *orientalis*, *F*. *moschata*, *F. bucharica*, *F. mandschurica*, and *F. vesca* in this clade. Nevertheless, it is difficult to conclude that *F*. *virdis* is the ancestor of the rest of the species in group D, for the lack of adequate within-species diversity samples. This can partially explaine why antecedent research did not support the *F. virdis* as one of the subgenomes of octoploid strawberry. The tetraploid species *F*. *orientalis* and hexaploid species *F. moschata* share the same female ancestor (**Figure 4**). Regarding the diploid species in 2x-6, *F. mandshurica* is related to *F. vesca*, which may occur gene introgression from *F. mandshurica* to *F*. *vesca* (Hummer et al., 2013). Haplotype network shows that *F. vesca* subsp. bracteate haplotype was the latest female donor to octoploid strawberry, which means hexaploid species *F. moschata* may contribute to the octoploid event.

### 4.3 Effects of the geographical distribution of wild species on *Fragaria* evolution

Wild *Fragaria* species are valuable resources for cultivated strawberry breeding improvement. The nucleotide diversity (π) of *Fragaria* chloroplast genomes shows low diversity of 8x accessions (**Figure 5B**). The overall lower Fst was obtained between 8x and other species (**Figure 5A**). These results suggested that wild species have significantly contributed to cultivated strawberry. Middle or East Asia was regarded as a center of diversity from which native diploid and tetraploid species spread (Staudt, 1999). According to the phylogenetic tree, species clustered into groups A, B, and C are native to Asia, especially China. There is little known about tetraploid species in group B. It is more likely that these species are limited to Asia, and excluded from the formation of octoploid. Haplotype network also supports this speculation (**Figure 4**).

Interspecific hybridization between *Fragaria* species with lower ploidy levels was used to develop gene introgression into octoploid cultivars (Bors and Sullivan, 2005). The distribution of species in group D and E are from Asia to Europe. *F*. *virids* is distributed in Asia and Europe, and partially overlaps with the hexaploid *F*. *moschata* natived to Europe (Edgar et al., 2018). *F*. *orientalis* is distributed from Asia to Eastern Siberia, and haplotypes in *F*. *orientalis* and *F*. *moschata* were closer relationships (**Figure 4**). *F*. *vesca* is the most widely distributed diploid. and *F*. *vesca* subsp. bracteata is native to North America (Staudt, 1999), which coincides with *F*. *chiloensis* and *F*. *virginiana* geographical distribution. Consequently, species in group D are most likely to have contributed to the formation of octoploid strawberry.

## Conclusion

In this study, we conducted a comparison of the chloroplast genomes assembled with short- and long-read data of *F. ananassa* cultivated species ‘Benihoppe’. we concluded that the chloroplast genome assemblies based on Illumina data were more accurate than CCS reads. We assembled 200 chloroplast genomes including 21 *Fragaria* species and outgroups. Based on sequence diversity, the phylogenetic tree and PCA analysis showed that *Fragaria* species could be divided into five groups. The *F*. *nilgerrensis* species form a single clade, in line with its unique morphological observation. Furthermore, we support that *F*. *vesca* subsp. bracteata was the last maternal donor to octoploid strawberry, which speculated that *F*. *moschata* may involve in the origin of octoploid strawberry.

## Data availability statement

The data presented in the study are deposited in the NCBI SRA (BioProject ID: PRJNA913463).

## Author contributions

YS and CL performed the research and analyzed the data. These authors contributed equally to this work and share the first authorship. HZ designed the chloroplast genomes research. LL, PH, GL, XZ and HZ contributed to the collection and conservation of wild resources. YS wrote the manuscript. All authors contributed to the article and approved the submitted version. All authors contributed to the article and approved the submitted version.

## Supplementary material

Supplementary Figure 1 Schematic diagram of sequence coverage of the *Fragaria ananassa* cv. Benihoppe chloroplast genome. The red line at the top of the schematic represents the ‘Benihoppe’ chloroplast genomes using Illumina data. The orange and bold blue lines indicate the contigs produced by Hifiasm and Canu software, respectively.

Supplementary Figure 2–7 The sequence alignment of PCR amplification and Sanger sequence of the obvious InDels I-VI in, respectively.
