# Supplementary figures and images for: The population genomic analyses of chloroplast genomes shed new insights on the complicated ploidy and evolutionary history in Fragaria
Source: Front Plant Sci. 2023 Feb 15;13:1065218. doi: 10.3389/fpls.2022.1065218 (PMC9975502; doi:10.3389/fpls.2022.1065218)

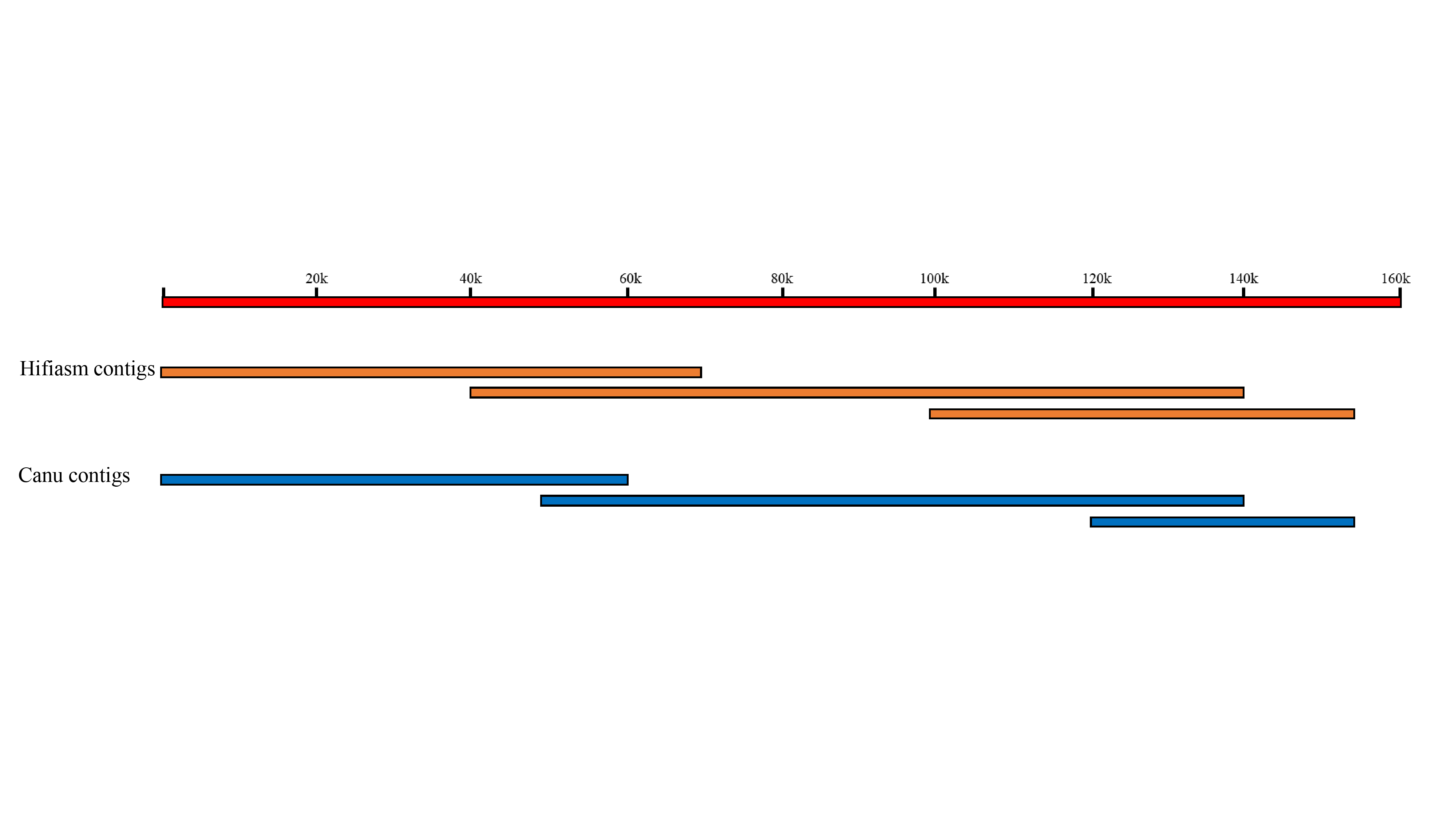

Supplement: Supplementary Figure 1 — Schematic diagram of sequence coverage of the Fragaria ananassa cv. Benihoppe chloroplast genome. The red line at the top of the schematic represents the ‘Benihoppe’ chloroplast genomes using Illumina data. The orange and bold blue lines indicate the contigs produced by Hifiasm and Canu software, respectively. [file Image_1.png]
