# Supplementary material for: The population genomic analyses of chloroplast genomes shed new insights on the complicated ploidy and evolutionary history in Fragaria
Source: Front Plant Sci. 2023 Feb 15;13:1065218. doi: 10.3389/fpls.2022.1065218 (PMC9975502; doi:10.3389/fpls.2022.1065218)
Supplement: Supplementary Figure 2–7 — The sequence alignment of PCR amplification and Sanger sequence of the obvious InDels I-VI in, respectively. [file Image_2.pdf]

```
Hifiasm_contig  ATCGAATTGCGTTTTTAGTTGCGGATGCGGAAAGGACAGAAGATACTTTGTATACATATT   60
Canu_contig     ATCGAATTGCGTTTTTAGTTGCGGA......AAGGACAGAAGATACTTTGTATACATATT   54
Illumina        ATCGAATTGCGTTTTTAGTTGCGGA......AAGGACAGAAGATACTTTGTATACATATT   54
Sanger          ATCGAATTGCGTTTTTAGTTGCGGA......AAGGACAGAAGATACTTTGTATACATATT   54
Consensus       atcgaattgcgtttttagttgcgga      aaggacagaagatactttgtatacatatt

Hifiasm_contig  CATCAAAGTCTTTGAGTACTCCGGTATTCAATCAATTCAATCAATATTAATTCGATTGAA   120
Canu_contig     CATCAAAGTCTTTGAGTACTCCGGTATTCAATCAATTCAATCAATATTAATTCGATTGAA   114
Illumina        CATCAAAGTCTTTGAGTACTCCGGTATTCAATCAATTCAATCAATATTAATTCGATTGAA   114
Sanger          CATCAAAGTCTTTGAGTACTCCGGTATTCAATCAATTCAATCAATATTAATTCGATTGAA   114
Consensus       catcaaagtctttgagtactccggtattcaatcaattcaatcaatattaattcgattgaa

Hifiasm_contig  TGAGTAATTGGCTTTTACTTATATATAATATATACCTTTTTTTATATAATATATACCTTT   180
Canu_contig     TGAGTAATTGGCTTTTACTTATATATAATATATACCTTTTTTC.................   157
Illumina        TGAGTAATTGGCTTTTACTTATATATAATATATACCTTTTTTC.................   157
Sanger          TGAGTAATTGGCTTTTACTTATATATAATATATACCTTTTTTC.................   157
Consensus       tgagtaattggcttttacttatatataatatataccttttt t

Hifiasm_contig  TTTCTTTTTTTTTTCGTTAACCTCTAGGCA   210
Canu_contig     ..TTTTTTTTTTTTCGTTAACCTCTAGGCA   185
Illumina        ..TTTTTTTTTTTTCGTTAACCTCTAGGCA   185
Sanger          ..TTTTTTTTTTTTCGTTAACCTCTAGGCA   185
Consensus         t tttttttttttcgttaacctctaggca
```
