# Supplementary material for: The population genomic analyses of chloroplast genomes shed new insights on the complicated ploidy and evolutionary history in Fragaria
Source: Front Plant Sci. 2023 Feb 15;13:1065218. doi: 10.3389/fpls.2022.1065218 (PMC9975502; doi:10.3389/fpls.2022.1065218)
Supplement: Supplementary file 4 [file Image_4.pdf]

```
Hifiasm_contig  ATAAGTTGATTCCTAACTTTTATCTCATATCATGACATAAGTAAGCAGTTCTTATTGTAT   60
Canu_contig     ATAAGTTGATTCCTAACTTTTATCTCATATCATGACATAAGTAAGCAGTTCT.ATTGTAT   59
Illumina        ATAAGTTGATTCCTAACTTTTATCTCATATCATGACATAAGTAAGCAGTTCTTATTGTAT   60
Sanger          ATAAGTTGATTCCTAACTTTTATCTCATATCATGACATAAGTAAGCAGTTCTTATTGTAT   60
Consensus       ataagttgattcctaacttttatctcatatcatgacataagtaagcagttct attgtat

Hifiasm_contig  CGGACCAAAACCTCGCGAATTGATCTTTACGGCGCTTCCTCTATCAAT.TAGATCCTTTA  119
Canu_contig     CGGACCAAAACCTCGCGAATTGATCTTTACGGCGCTTCCTCTATCAATATAGATCCTTTA  119
Illumina        CGGACCAAAACCTCGCGAATTGATCTTTACGGCGCTTCCTCTATCAAT.TAGATCCTTTA  119
Sanger          CGGACCAAAACCTCGCGAATTGATCTTTACGGCGCTTCCTCTATCAAT.TAGATCCTTTA  119
Consensus       cggaccaaaacctcgcgaattgatctttacggcgcttcctctatcaat tagatccttta

Hifiasm_contig  TCCATAGAATAAAGTATTTAGGCATACCTATTTCTTCATATTAATATTTCGACTTTTATG  179
Canu_contig     TCCATAGAATAAAGTATTTAGGCATACCTATTTCTTCATATTAATATTTCGACTTTTATG  179
Illumina        TCCATAGAATAAAGTATTTAGGCATACCTATTTCTTCATATTAATATTTCGACTTTTATG  179
Sanger          TCCATAGAATAAAGTATTTAGGCATACCTATTTCTTCATATTAATATTTCGACTTTTATG  179
Consensus       tccatagaataaagtatttaggcatacctatttcttcatattaatatttcgacttttatg

Hifiasm_contig  AAGTTTATTTCCTTGCTACAGCTGATAAAAATCGTTGT.TTTGAACGATACATATGTAGA  238
Canu_contig     AAGTTTATTTCCTTGCTACAGCTGATAAAAATCGTTGTGTTTGAACGATACATATGTAGA  239
Illumina        AAGTTTATTTCCTTGCTACAGCTGATAAAAATCGTTGT.TTTGAACGATACATATGTAGA  238
Sanger          AAGTTTATTTCCTTGCTACAGCTGATAAAAATCGTTGT.TTTGAACGATACATATGTAGA  238
Consensus       aagtttatttccttgctacagctgataaaaatcgttgt tttgaacgatacatatgtaga

Hifiasm_contig  AAGCCCACCTTCTTTTTGTATT.......................TATTAATATAATATT  275
Canu_contig     AAGCCTACCTTCATTTTGTATTTATTAATATAATATTTTTTTATTTATTAATATAATATT  299
Illumina        AAGCCTACCTTCTTTTTGTATTTATTAATATAATATTTTTT.ATTTATTAATATAATATT  297
Sanger          AAGCCTACCTTCTTTTTGTATTTATTAATATAATATTTTTT.ATTTATTAATATAATATT  297
Consensus       aagcc accttc ttttgtatt                       tattaatataatatt

Hifiasm_contig  TTTATTAAATTAATATTTTAATATTATAGTATTTATTAACGGATTTCTTCCCCTTTTTTT  335
Canu_contig     TTTATTAAATTAATATTTTAATATTATAGTATTTAATAACGGATTTCTTCCCCTTTTTTT  359
Illumina        TTTATTAAATTAATATTTTAATATTATAGTATTTATTAACGGATTTCTTCCCCTTTTTTT  357
Sanger          TTTATTAAATTAATATTTTAATATTATAGTATTTATTAACGGATTTCTTCCCCTTTTTTT  357
Consensus       tttattaaattaatattttaatattatagtattta taacggatttcttcccctttttttt

Hifiasm_contig  ATTTCTATAGTGGAGATAGTCGCACGTAATGACAGATCACGGCCATATTATTAAAAGCTT  395
Canu_contig     ATTTCTATAGTGGAGATAGTCGCACGTAATGACAGATCACGGCCATATTATTAAAAGCTT  419
Illumina        ATTTCTATAGTGGAGATAGTCGCACGTAATGACAGATCACGGCCATATTATTAAAAGCTT  417
Sanger          ATTTCTATAGTGGAGATAGTCGCACGTAATGACAGATCACGGCCATATTATTAAAAGCTT  417
Consensus       atttctatagtggagatagtcgcacgtaatgacagatcacggccatattattaaaagctt

Hifiasm_contig  GTGGTAAGAACGGGTTTC                                            413
Canu_contig     GTGGTAAGAACGGGTTTC                                            437
Illumina        GTGGTAAGAACGGGTTTC                                            435
Sanger          GTGGTAAGAACGGGTTTC                                            435
Consensus       gtggtaagaacgggtttc
```
