# Supplementary material for: The population genomic analyses of chloroplast genomes shed new insights on the complicated ploidy and evolutionary history in Fragaria
Source: Front Plant Sci. 2023 Feb 15;13:1065218. doi: 10.3389/fpls.2022.1065218 (PMC9975502; doi:10.3389/fpls.2022.1065218)
Supplement: Supplementary file 5 [file Image_5.pdf]

```
Hifiasm_contig  AAAATACCAAGTAAACCAATTACAACAATACCGGTTACAGTACCTATTATCCAAAGAGGA  60
Canu_contig     AAAATACCAAGTAAACCAATTACAACAATACCGGTTACAGTACCTATTATCCAAAGAGGA  60
Illumina        AAAATACCAAGTAAACCAATTACAACAATACCGGTTACAGTACCTATTATCCAAAGAGGA  60
Sanger          AGAATACCAATTAAATCAATTACAACAATACCGGTTACAGTACCTATTATCCAAAGAGGA  60
Consensus       a aataccaa taaa caattacaacaataccggttacagtacctattatccaaagagga

Hifiasm_contig  ATCCTTCCAGTAGTATCGGCCATTTGCCCGACTTTCCTCCACATTTTTAAAGTGGTCATG  120
Canu_contig     ATCCTTCCAGTAGTATCGGCCATTTGCCCGACTTTCCTCCACATTTTTAAAGTGGTCATG  120
Illumina        ATCCTTCCAGTAGTATCGGCCATTTGCCCGACTTTCCTCCACATTTTTCAAGTGGTCATG  120
Sanger          ATCCTTCCAGTAGTATCGGCCATTTGCCCGACTTTCCTCCACATTTTTCAAGTGGTCATG  120
Consensus       atccttccagtagtatcggccatttgcccgactttcctccacattttt aagtggtcatg

Hifiasm_contig  CTAGAGACATAAACAGCCATAGATAATTATGAGATGATATCTTTCCGAATGGGATAAGAG  180
Canu_contig     CTAGAGACATAAACAGCCATAGATAATTATGAGATGATATCTTTCCGAATGGGATAAGAG  180
Illumina        CTAGAGACATAAACAGCCATAGATAATTATGAGATGATATCTTTCCGAATGGGATAAGAG  180
Sanger          CTAGAGACATAAACAGCCATAGATAATTATGAGATGATATCTTTCCGAATGGGATAAGAA  180
Consensus       ctagagacataaacagccatagataattatgagatgatatctttccgaatgggataaga

Hifiasm_contig  AATTCCTACTATATAT.........TAGTAGTATATATTATTTTTGTATTAATTAAAGAA  231
Canu_contig     AATTCCTACTATATAT.........TAGTAGTATATATTATTTTTGTATTAATTAAAGAA  231
Illumina        AATTCCTACTATATATACTATATATTAGTAGTATATATTATTTTTGTATTAATTAAAGAA  240
Sanger          AATTCCTACTATATAT.........TAGTAGTATATATTATTTTTGTATTAATTAAAGAA  231
Consensus       aattcctactatatat         tagtagtatatattatttttgtattaattaaagaa

Hifiasm_contig  ATAATTGGAAAATAAAACAGCAAGTACAAAAATGAGTAATAACCCCCAGTAGAGACTGGT  291
Canu_contig     ATAATTGGAAAATAAAACAGCAAGTACAAAAATGAGTAATAACCCCCAGTAGAGACTGGT  291
Illumina        ATAATTGGAAAATAAAACAGCAAGTACAAAAATGAGTAATAACCCCCAGTAGAGACTGGT  300
Sanger          ATAATTGGAAAATAAAACAGCAAGTACAAAAATGAGTAATAACCCCAAGTAGAGACTGGT  291
Consensus       ataattggaaaataaaacagcaagtacaaaaatgagtaataacccc agtagagactggt

Hifiasm_contig  ACGATTCAATTCAACACTTTGTTCGTTCGGGTTTGATTGTGTCATAGCTCTATGATTCGG  351
Canu_contig     ACGATTCAATTCAACACTTTGTTCGTTCGGGTTTGATTGTGTCATAGCTCTATGATTCGG  351
Illumina        ACGATTCAATTCAACACTTTGTTCGTTCGGGTTTGATTGTGTCATAGCTCTATGATTCGG  360
Sanger          ACGATTCAATTCAACACTTTGTTCATTCGGGTTTGATTGTGTCATAGCTCTATGATTCGG  351
Consensus       acgattcaattcaacactttgttc ttcgggtttgattgtgtcatagctctatgattcgg

Hifiasm_contig  ATTAGGTTTATCGTTGGATGAACTGCATTGCGGATATTGACCCTAAAAAAAAAACGGTAG  411
Canu_contig     ATTAGGTTTATCGTTGGATGAACTGCATTGCGGATATTGACCCTAAAAAAAAAACGGTAG  411
Illumina        ATTAGGTTTATCGTTGGATGAACTGCATTGCGGATATTGACCCTAAAAAAAAAACGGTAG  420
Sanger          ATTAGGTTTATTGTTGGATGAACTGCATTGCGGATATTGACCCCAAAAAAAAA.CGGTAG  410
Consensus       attaggtttat gttggatgaactgcattgcggatattgaccc aaaaaaaaa cggtag

Hifiasm_contig  GTACAGCTAGTCCGTGAACCGCTAACCATCGTACTGTAAAAATCGGATAGGTTCTATCTA  471
Canu_contig     GTACAGCTAGTCCGTGAACCGCTAACCATCGTACTGTAAAAATCGGATAGGTTCTATCTA  471
Illumina        GTACAGCTAGTCCGTGAACCGCTAACCATCGTACTGTAAAAATCGGATAGGTTCTATCTA  480
Sanger          GTACAGCTAGTCCGTGAACCGCTAACCATCGTACTGTAAAAATCAGATAGGTTCTATCTA  470
Consensus       gtacagctagtccgtgaaccgctaaccatcgtactgtaaaaatc gataggttctatcta

Hifiasm_contig  TAGTCATTGGGGCCTCCTAAAAAGATCTGCTAAATTCATCGAGTTGTTCCAAAGGATCAA  531
Canu_contig     TAGTCATTGGGGCCTCCTAAAAAGATCTGCTAAATTCATCGAGTTGTTCCAAAGGATCAA  531
Illumina        TAGTCATTGGGGCCTCCTAAAAAGATCTGCTAAATTCATCGAGTTGTTCCAAAGGATCAA  540
Sanger          TAGTCATTGGGGCCTCCTAAAAAGATTTGCTAAATTCATCTAGTTGTTCCAAAGGATCAA  530
Consensus       tagtcattggggcctcctaaaaagat tgctaaattcatc agttgttccaaaggatcaa

Hifiasm_contig  AACGGCCAGTTATTAAGGGAATTCCTTGTCGGCTTTCTGTAAAATACTCATTTGGACGGG  591
Canu_contig     AACGGCCAGTTATTAAGGGAATTCCTTGTCGGCTTTCTGTAAAATACTCATTTGGACGGG  591
Illumina        AACGGCCAGTTATTAAGGGAATTCCTTGTCGGCTTTCTGTAAAATACTCATTTGGACGGG  600
Sanger          AACGGCCAGTTATTAAGGGAATTCCTTGTCGGCTTTCTGTAAAATACTCATTTGGACGGG  590
Consensus       aacggccagttattaagggaattccttgtcggctttctgtaaaatactcatttggacggg

Hifiasm_contig  GGCTTCCAAATACATCGTAAGCTAAACCAGTGCTGACGAATAACCAACCCGCAATAAATA  651
Canu_contig     GGCTTCCAAATACATCGTAAGCTAAACCAGTGCTGACGAATAACCAACCCGCAATAAATA  651
Illumina        GGCTTCCAAATACATCGTAAGCTAAACCAGTGCTGACGAATAACCAACCCGCAATAAATA  660
Sanger          GGCTTCCAAATACATAGTAAGCTAAACCAGTGCTGACGAATAACCAACCCGCAATAAATA  650
Consensus       ggcttccaaatacat gtaagctaaaccagtgctgacgaataaccaacccgcaataaata

Hifiasm_contig  GGGAAGGTATAGTAATGCTATGAATGACCCAGTATCGGATACTGGTAATAATATCAGCAA  711
Canu_contig     GGGAAGGTATAGTAATGCTATGAATGACCCAGTATCGGATACTGGTAATAATATCAGCAA  711
Illumina        GGGAAGGTATAGTAATGCTATGAATGACCCAGTATCGGATACTGGTAATAATATCAGCAA  720
Sanger          GGGAAGGTATAGTAATGCTATGAATGACCCAGTATCGGATACTGGTAATAATATCAGCAA  710
Consensus       gggaaggtatagtaatgctatgaatgacccagtatcggatactggtaataatatcagcaa

Hifiasm_contig  AAGAACGTTCTCCTGTGCTTCCAGACATGTTGAGCT                          747
Canu_contig     AAGAACGTTCTCCTGTGCTTCCAGACATGTTGAGCT                          747
Illumina        AAGAACGTTCTCCTGTGCTTCCAGACATGTTGAGCT                          756
Sanger          AAGAACGTTCTCCTGTGCTTCCAGACATGTTGAGCT                          746
Consensus       aagaacgttctcctgtgcttccagacatgttgagct
```
