# Supplementary material for: The population genomic analyses of chloroplast genomes shed new insights on the complicated ploidy and evolutionary history in Fragaria
Source: Front Plant Sci. 2023 Feb 15;13:1065218. doi: 10.3389/fpls.2022.1065218 (PMC9975502; doi:10.3389/fpls.2022.1065218)
Supplement: Supplementary file 6 [file Image_6.pdf]

```
Hifiasm_contig  ATGGCCTTACTTTTTCTTTCTTTTTATTTATTTATTATTTCTCGTTAATGCGGGTTAATT  60
Canu_contig     ATGGCCTTACTTTTTCTTTCTTTTTATTTATTTATTATTTCTCGTTAATGCGGGTTAATT  60
Illumina        ATGGCCTTACTTTTTCTTTCTTTTTATTTA....TTATTTCTCGTTAATGCGGGTTAATT  56
Sanger          ATGGCCTTACTTTTTCTTTCTTTTTATTTA....TTATTTCTCGTTAATGCGGGTTAATT  56
Consensus       atggccttactttttctttctttttattta    ttatttctcgttaatgcgggttaatt

Hifiasm_contig  AAGTATAGTATGCCCGGCACGAATCAGCATATGTTTTACGCCCCGTAATTCTTCCTCAGC  120
Canu_contig     AAGTATAGTATGCCCGGCACGAATCAGCATATGTTTTACGCCCCGTAATTCTTCCTCAGC  120
Illumina        AAGTATAGTATGCCCGGCACGAATCAGCATATGTTTTACGCCCCGTAATTCTTCCTCAGC  116
Sanger          AAGTATAGTATGCCCGGCACGAATCAGCATATGTTTTACGCCCCGTAATTCTTCCTCAGC  116
Consensus       aagtatagtatgcccggcacgaatcagcatatgttttacgccccgtaattcttcctcagc

Hifiasm_contig  CAGGCTGGGGCAGAATAGCAGAGCAAGTACAAGTATTATATTAGTTATTAGTAGCATAGC  180
Canu_contig     CAGGCTGGGGCAGAATAGCAGAGCAAGTACAAGTATTATATTAGTTATTAGTAGCATAGC  180
Illumina        CAGGCTGGGGCAGAATAGCAGAGCAAGTACAAGTATTATATTAGTTATTAGTAGCATAGC  176
Sanger          CAGGCTGGGGCAGAATAGCAGAGCAAGTACAAGTATTATATTAGTTATTAGTAGCATAGC  176
Consensus       caggctggggcagaatagcagagcaagtacaagtattatattagttattagtagcatagc

Hifiasm_contig  AAAAATGCGTTCCTCGTCATTCGCCGAGGTATTGACGGTGATTCTCAGCAGAACAGAATG  240
Canu_contig     AAAAATGCGTTCCTCGTCATTCGCCGAGGTATTGACGGTGATTCTCAGCAGAACAGAATG  240
Illumina        AAAAATGCGTTCCTCGTCATTCGCCGAGGTATTGACGGTGATTCTCAGCAGAACAGAATG  236
Sanger          AAAAATGCGTTCCTCGTCATTCGCCGAGGTATTGACGGTGATTCTCAGCAGAACAGAATG  236
Consensus       aaaaatgcgttcctcgtcattcgccgaggtattgacggtgattctcagcagaacagaatg

Hifiasm_contig  TAATACGATGAGATAGAATGCAATAGAAACAAAGACACAGGGAACGGGTTACCTACTCTT  300
Canu_contig     TAATACGATGAGATAGAATGCAATAGAAACAAAGACACAGGGAACGGGTTACCTACTCTT  300
Illumina        TAATACGATGAGATAGAATGCAATAGAAACAAAGACACAGGGAACGGGTTACCTACTCTT  296
Sanger          TAATACGATGAGATAGAATGCAATAGAAACAAAGACACAGGGAACGGGTTACCTACTCTT  296
Consensus       taatacgatgagatagaatgcaatagaaacaaagacacagggaacgggttacctactctt

Hifiasm_contig  AACGGTCAAA  310
Canu_contig     AACGGTCAAA  310
Illumina        AACGGTCAAA  306
Sanger          AACGGTCAAA  306
Consensus       aacggtcaaa
```
