# Supplementary material for: The population genomic analyses of chloroplast genomes shed new insights on the complicated ploidy and evolutionary history in Fragaria
Source: Front Plant Sci. 2023 Feb 15;13:1065218. doi: 10.3389/fpls.2022.1065218 (PMC9975502; doi:10.3389/fpls.2022.1065218)
Supplement: Supplementary file 7 [file Image_7.pdf]

```
Hifiasm_contig  TGACTGGGCGGTAGAGTCGTGGAAACAACACCTGTTTCTTCCATATTTTGGACCTTAGCT  60
Canu_contig     TGACTGGGCGGTAGAGTCGTGGAAACAACACCTGTTTCTTCCATATTTTGGACCTTAGCT  60
Illumina        TGACTGGGCGGTAGAGTCGTGGAAA...CACCTGTTTCTTCCATATTTTGGACCTTAGCT  57
Sanger          TGACTGGGCGGTAGAGTCGTGGAA....CACCTGTTTCTTCCATATTTTGGACCTTAGCT  56
Consensus       tgactgggcggtagagtcgtggaa    cacctgtttcttccatattttggaccttagct

Hifiasm_contig  CCATGGAACAATATACTAACTACTGCTGAAACATGGAAGAATTGAAATCTTAGATCAAAA  120
Canu_contig     CCATGGAACAATATACTAACTACTGCTGAAACATGGAAGAATTGAAATCTTAGATCAAAA  120
Illumina        CCATGGAACAATATACTAACTACTGCTGAAACATGGAAGAATTGAAATCTTAGATCAAAA  117
Sanger          CCATGGAACAATATACTAACTACTGCTGAAACATGGAAGAATTGAAATCTTAGATCAAAA  116
Consensus       ccatggaacaatatactaactactgctgaaacatggaagaattgaaatcttagatcaaaa

Hifiasm_contig  CATTATGTATGGATGGTATGAACTGCCTAAACAAGAATTCTTGAACAGCGAACAACCGGA  180
Canu_contig     CATTATGTATGGATGGTATGAACTGCCTAAACAAGAATTCTTGAACAGCGAACAACCGGA  180
Illumina        CATTATGTATGGATGGTATGAACTGCCTAAACAAGAATTCTTGAACAGCGAACAACCGGA  177
Sanger          CATTATGTATGGATGGTATGAACTGCCTAAACAAGAATTCTTGAACAGCGAACAACCGGA  176
Consensus       cattatgtatggatggtatgaactgcctaaacaagaattcttgaacagcgaacaaccgga

Hifiasm_contig  GCTATTACTCACTACATCAAAAAATTTCCATTAATGAAAGATGTAAATCCATTGGAAAAT  240
Canu_contig     GCTATTACTCACTACATCAAAAAATTTCCATTAATGAAAGATGTAAATCCATTGGAAAAT  240
Illumina        GCTATTACTCACTACATCAAAAAATTTCCATTAATGAAAGATGTAAATCCATTGGAAAAT  237
Sanger          GCTATTACTCACTACATCAAAAAATTTCCATTAATGAAAGATGTAAATCCATTGGAAAAT  236
Consensus       gctattactcactacatcaaaaaatttccattaatgaaagatgtaaatccattggaaaat

Hifiasm_contig  CAAAAATACGCATGTTGGATGAAATGGTTGTTGCTATCTGCTACAATAACGACTCGTTGG  300
Canu_contig     CAAAAATACGCATGTTGGATGAAATGGTTGTTGCTATCTGCTACAATAACGACTCGTTGG  300
Illumina        CAAAAATACGCATGTTGGATGAAATGGTTGTTGCTATCTGCTACAATAACGACTCGTTGG  297
Sanger          CAAAAATACGCATGTTGGATGAAATGGTTGTTGCTATCTGCTACAATAACGACTCGTTGG  296
Consensus       caaaaatacgcatgttggatgaaatggttgttgctatctgctacaataacgactcgttgg

Hifiasm_contig  TTTAACTGAATAACTAAATAAAATAGATAGACATTTCTCTTCGTCTCAGGTCGACGGATC  360
Canu_contig     TTTAACTGAATAACTAAATAAAATAGATAGACATTTCTCTTCGTCTCAGGTCGACGGATC  360
Illumina        TTTAACTGAATAACTAAATAAAATAGATAGACATTTCTCTTCGTCTCAGGTCGACGGATC  357
Sanger          TTTAACTGAATAACTAAATAAAATAGATAGACATTTCTCTTCGTCTCAGGTCGACGGATC  356
Consensus       tttaactgaataactaaataaaatagatagacatttctcttcgtctcaggtcgacggatc

Hifiasm_contig  TTCTCAATTGAAAGACCCCCTATATGGATAATACACATTCCAGTTGACCGACTAATTCGA  420
Canu_contig     TTCTCAATTGAAAGACCCCCTATATGGATAATACACATTCCAGTTGACCGACTAATTCGA  420
Illumina        TTCTCAATTGAAAGACCCCCTATATGGATAATACACATTCCAGTTGACCGACTAATTCGA  417
Sanger          TTCTCAATTGAAAGACCCCCTATATGGATAATACACATTCCAGTTGACCGACTAATTCGA  416
Consensus       ttctcaattgaaagaccccctatatggataatacacattccagttgaccgactaattcga

Hifiasm_contig  ATTGTTTTGTTCCGAAGCAAAGATATCCGCGGGGCGGTTCGTCCTATTCAGATATTCACG  480
Canu_contig     ATTGTTTTGTTCCGAAGCAAAGATATCCGCGGGGCGGTTCGTCCTATTCAGATATTCACG  480
Illumina        ATTGTTTTGTTCCGAAGCAAAGATATCCGCGGGGCGGTTCGTCCTATTCAGATATTCACG  477
Sanger          ATTGTTTTGTTCCGAAGCAAAGATATCCGCGGGGCGGTTCGTCCTATTCAGATATTCACG  476
Consensus       attgttttgttccgaagcaaagatatccgcggggcggttcgtcctattcagatattcacg

Hifiasm_contig  ACCAAGAAGTACTGGATTCTCTTTCGGGTAGGCCCTGAAAGGAGAAGGAAGGCTGGAATG  540
Canu_contig     ACCAAGAAGTACTGGATTCTCTTTCGGGTAGGCCCTGAAAGGAGAAGGAAGGCTGGAATG  540
Illumina        ACCAAGAAGTACTGGATTCTCTTTCGGGTAGGCCCTGAAAGGAGAAGGAAGGCTGGAATG  537
Sanger          ACCAAGAAGTACTGGATTCTCTTTCGGGTAGGCCCTGAAAGGAGAAGGAAGGCTGGAATG  536
Consensus       accaagaagtactggattctctttcgggtaggccctgaaaggagaaggaaggctggaatg

Hifiasm_contig  CCAACGGGCGTCTATTATTGAATTATTGAATTCACCCGACCCGATAGTACCCATTTTGGG  600
Canu_contig     CCAACGGGCGTCTATTATTGAATTATTGAATTCACCCGACCCGATAGTACCCATTTTGGG  600
Illumina        CCAACGGGCGTCTATTATTGAATT........CACCCGACCCGATAGTACCCATTTTGGG  589
Sanger          CCAACGGGCGTCTATTATTGAATT........CACCCGACCCGATAGTACCCATTTTGGG  588
Consensus       ccaacgggcgtctattattgaatt        cacccgacccgatagtacccattttggg

Hifiasm_contig  AACGTCCAGTGCCAAAGTCACTGAATGGGTAAGTCCCCAATCCCTAAAACGGACTATGTA  660
Canu_contig     AACGTCCAGTGCCAAAGTCACTGAATGGGTAAGTCCCCAATCCCTAAAACGGACTATGTA  660
Illumina        AACGTCCAGTGCCAAAGTCACTGAATGGGTAAGTCCCCAATCCCTAAAACGGACTATGTA  649
Sanger          AACGTCCAGTGCCAAAGTCACTGAATGGGTAAGTCCCCAATCCCTAAAACGGACTATGTA  648
Consensus       aacgtccagtgccaaagtcactgaatgggtaagtccccaatccctaaaacggactatgta

Hifiasm_contig  ATGTACTT  668
Canu_contig     ATGTACTT  668
Illumina        ATGTACTT  657
Sanger          ATGTACTT  656
Consensus       atgtactt
```
